# Impact of Divergent Thinking Training on Teenagers’ Emotion and Self-Efficacy During the COVID-19 Pandemic

**DOI:** 10.3389/fpsyg.2021.600533

**Published:** 2021-03-19

**Authors:** Bin Zuo, Qi Wang, Yalan Qiao, Yu Ding, Fangfang Wen

**Affiliations:** Department of Psychology, Center for Studies of Social Psychology, Central China Normal University, Wuhan, China

**Keywords:** divergent thinking, COVID-19, self-efficacy, emotion, teenagers

## Abstract

Currently due to the COVID-19 pandemic, young people are experiencing a decrease in self-efficacy and an increase in mental illness. Though previous studies have shown that self-efficacy and divergent thinking training are positively related, little is known about the impact of divergent thinking training on self-efficacy and emotions. Therefore, our study seeks this answer to support teenagers injured psychologically during disastrous periods. We randomly assigned 70 students to a 2 (time: pretest, post-test) × 2 (groups: divergent thinking training, controlled) mixed design. Participants in the experimental group were given a 9-day divergent thinking training with the theme of “writing down 10 novel functions of the mask,” while those in the control group spent 10 min each day recording what they ate. The self-efficacy, anxiety, depression, and stress of two groups were measured before and after training. Results showed that, compared to the control group, self-efficacy ceased decreasing while anxiety decreased for the experimental group. These findings confirm the positive effect of divergent thinking on teenagers. Implications and limitations are discussed.

## Introduction

The outbreak of the novel coronavirus disease 2019 (COVID-19) began in December 2019 in Wuhan, Hubei, China. On January 30, 2020, it was declared a public health emergency of international concern by the World Health Organization (National health commision of the People’s Republic of China, 2020b). COVID-19 is highly infectious and can be transmitted via respiratory droplets and close contact (National health commision of the People’s Republic of China, 2020c). Most infected patients have exhibited fever, cough, headache, and other symptoms (Huang et al., 2020). Through August 19, 2020, China is still in the pandemic period, with 84,895 confirmed cases and 4,634 deaths (National health commision of the People’s Republic of China, 2020d).

Due to the COVID-19 pandemic, teenagers have experienced different degrees of psychologically adverse reactions. By conducting an online survey, researchers found that people aged 12–21.4 years scored higher on the IES-R scale (measuring PTSD symptoms) than those aged 49.6–59 years (Wang et al., 2020). Similarly, Duan et al. (2020) conducted a survey among young people aged 7–18 years in mainland China, and the results demonstrated that adolescents (aged 13–18 years) exhibited higher degrees of anxiety than children (aged 7–12 years). Furthermore, their research showed that children and adolescents suffered more clinical depression symptoms, reaching 22.28% during the COVID-19 pandemic, compared to 13.2% when measured during a normal time (Stewart and Sun, 2007).

Self-efficacy, a belief in one’s ability to achieve a desirable outcome, was proposed by Bandura (1977). Several studies have proved that self-efficacy is effective in reducing stress and generating coping strategies for individuals facing stressful life events (Saccinto et al., 2013; Marceron and Rohrbeck, 2019; Ringeisen et al., 2019). During the COVID-19 pandemic, self-efficacy has played a positive role in maintaining optimism and mental health (Garris and Fleck, 2020; Kebede et al., 2020; Tabernero et al., 2020). Garris and Fleck (2020) found that when required to study online during COVID-19 period, students with high self-efficacy evaluated more positive about online courses. Despite of positive role of self-efficacy in coping with stressful events, those events in turn could damage self-efficacy. Saigh et al. (1995) conducted a research on adolescents aged 13, and found that those with PTSD had lower self-efficacy. A total of 393 Israeli veterans completed several questionnaires conducted by Ginzburg et al. (2003); the results showed that those injured in war had low self-efficacy. Alexander and Ward (2018) explained that people facing disasters exhibit low coping ability and physiological responses (i.e., sweat and pain), which reduces self-efficacy.

Terror management theory (Greenberg et al., 1986; Pyszczynski et al., 1990) posited that individuals are vulnerable to external threat. However, people can minimize terror and protect their mental health by maintaining self-esteem (self-efficacy). Anxiety, specifically, can be lowered accompanied by the maintenance of self-efficacy. The finding of Garnefski et al. (2002) demonstrated that teenagers are more vulnerable to psychological problems facing disasters, because their coping strategies are fewer compared to adults, which manifests as more rumination and less positive reappraisal of situations. Thus, it is imperative to support young people during this tough period and to rebuild their mental health.

### Divergent Thinking and Emotions

A variety of theories and models have posited that cognition drives emotion. According to Ellis’ ABC theory (Ellis, 1984, 1991), what a person believes or how he/she interprets external events ultimately results in his/her corresponding emotions and behaviors. This implies that cognition mediates between the activating event and the recipient’s reactions. Bar (2009) proposed that associative processing promotes positive emotions by preventing rumination. He explained that associations enable people to envision what could happen in the future, thereby alleviating negative emotions brought about by uncertainty. Among cognitive processes, divergent thinking, which is thinking from diverse directions (Guilford, 1956), is generally used to measure potential in creativity (Runco, 2017). Previous studies have shown that divergent thinking influences emotions: Akbari Chermahini and Hommel (2012) found the positive effect of divergent thinking on emotions, while Liknaitzky et al. (2018) showed that lack of divergent thinking or cognitive rigidity was correlated with depression. However, gaps in research remain and require further study. First, these studies were cross-sectional, leaving the long-term effect of divergent thinking unexamined. Second, these studies did not examine how divergent thinking exerts effect on specific emotion. Instead, they merely identified the correlation between divergent thinking and emotions, and tested the effect of divergent thinking on general emotions. Third, materials selected previously were not used to target a specific issue. For example, researchers merely asked participants to list various uses for items, which could be replaced by other items without affecting results. This study hypothesizes that people’s negative emotions (i.e., depression, stress, and anxiety) caused by the COVID-19 pandemic can be eliminated or alleviated through repeatedly disrupting negative association with masks.

More specifically, a mask currently represents the presence of the virus, social distance, and alienation from strangers. Thus, asking people to formulate other functions for a mask is assumed to effectively prevent negative attitudes toward a mask as well as toward the virus; this protects their mental state. Furthermore, divergent thinking training enables people to divert attention away from focusing on the negativity about COVID-19 spread by news and media, as ruminative thought is a confirmed predictor of emotion disorders (Smith and Alloy, 2009; Wilkinson et al., 2013; Ruscio et al., 2015).

### Divergent Thinking and Self-Efficacy

Previous studies that investigated the relationship between divergent thinking and self-efficacy mainly focused on how the latter impacts the former. Kharkhurin (2017) illustrated that creative self-efficacy (belief about one’s creative ability) contributed positively to divergent thinking by conducting tests on divergent thinking, fluid intelligence, and creative self-efficacy. Similarly, Puente-Díaz and Cavazos-Arroyo (2017) conducted several tests including divergent thinking, creative self-efficacy, and imagination, drawing the same conclusion as Kharkhurin. Besides the confirmed effect of creative self-efficacy on divergent thinking, some studies examined how creativity training promoted creative self-efficacy (Mathisen and Bronnick, 2009; Byrge and Tang, 2015). For instance, Meinel et al. (2019) conducted creativity training lasting several months and found enhancement of creative self-efficacy. Similarly, Byrge and Tang (2015) implemented a complex creativity training program that consists of five sections, and the results showed increase in both creative self-efficacy and creativity ability. In summary, these studies examined the positive effect of creative self-efficacy on divergent thinking and the positive effect of creativity training on creative self-efficacy. However, whether divergent thinking impacts general self-efficacy remains unknown. According to social cognitive theory, an external stimulus impacts self-efficacy via cognitive appraisal; hence, self-efficacy reflects how threatening an event is compared to an individual’s coping ability (Bandura, 1977; Benight and Bandura, 2004). Bandura (1977) and Benight and Bandura (2004) proposed that self-efficacy could be improved by lowering the perception of threat, which allows for the promotion of self-efficacy through divergent thinking. Accordingly, we believe that attaching various functions to masks decreases the perceived threat associated with masks and increases self-efficacy.

In our study, we adopt divergent thinking training, asking participants to list as many uses for masks as possible. We investigate whether divergent thinking exerts a positive effect on teenagers’ self-efficacy and emotions. Based on Ellis’ ABC theory, social cognitive theory, terror management theory, and previous studies, we hypothesize that participants’ self-efficacy will increase, while negative emotions (i.e., anxiety, stress, and depression) will decrease after divergent thinking training.

## Materials and Methods

### Participants

Before the study, we used G^∗^Power3.1 (Faul et al., 2007) software to estimate the sample size required for the study. Assuming that a moderate effect was obtained, *f* = 0.25, and power = 0.95, it would take at least 54 participants to detect significant intra-and inter-group interactions in the repeated measurement analysis of variance, that is, 27 in each group.

**FIGURE 1:**
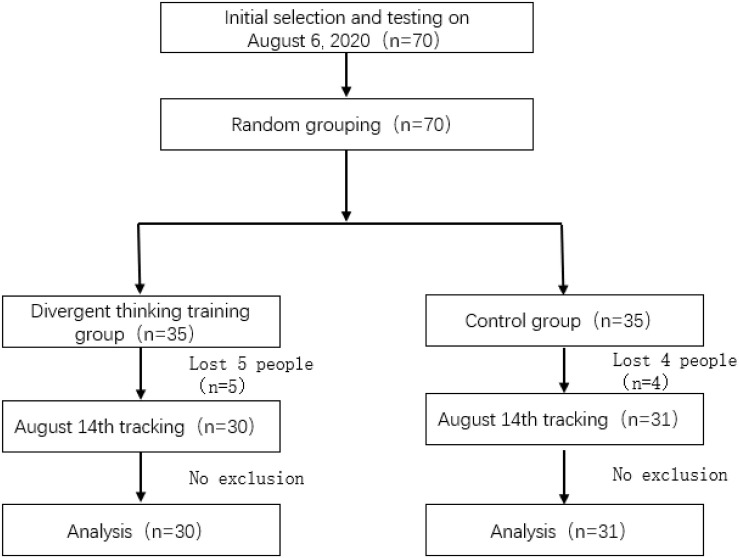
The illustration of the procedure of this study.

Figure 1 shows the procedure and the number of participants recruited in each phase of this study. At the beginning of this study, 70 participants were recruited by three experimenters based on random sampling. The participants were all from a randomly selected middle school in Wuhan who volunteered for this study. All participants were randomly assigned to an experimental group (divergent thinking group) (*n* = 35) and a control group (*n* = 35). They completed the background information questionnaire, the Self-Efficacy Scale, and the Depression-Anxiety-Stress Scale (T1) on August 6, 2020. Afterward, the experimental group completed a divergent thinking training task, while the control group completed a “writing down what to eat” task. Nine days after the end of the intervention (August 14, T2), all participants completed the post-test to assess their self-efficacy, anxiety, depression, and stress levels.

In the post-test, five participants dropped out in the divergent thinking training group and four participants in the control group. Thus, 61 participants came into analysis, with 30 in the divergent thinking training group and 31 in the control group. This number of participants met the basic needs of sample size.

During the pandemic, to avoid exposure to virus, the whole study was conducted online by three experimenters (graduate students). In this study, a single-blind setting was adopted; none of the participants had any knowledge of the distribution and purpose of the study, and none had participated in similar studies before. The procedure of this study was reviewed and approved online by the Ethics Review Committee of our community. Due to the quarantine protocols imposition, it was not convenient to print out and sign a written informed consent form, so all participants provided online oral informed consent before the test.

#### Intervention Procedure

During the intervention process, the divergent thinking training group was asked to list 10 novel functions for the mask. The instructions were as follows: “Please write down 10 novel functions for the mask (please fill in as much as possible within 10 min) (hint: the system will automatically jump to the next page in 10 min. Thank you for your participation).” The control group was asked to write down 10 kinds of food eaten that day. The instructions were as follows: “Please write down the food you ate today (please fill in as much as possible within 10 min) (hint: the system will automatically jump to the next page in 10 min. Thank you for your participation).” Considering the end time of the three meals for middle-school students, the training started at 8 pm every day, and the entire training lasted 9 days. All operations were carried out using an online questionnaire due to pandemic isolation. The minimum filling time of the questionnaire was 3 min, and the longest filling time was 10 min, for the purpose of carefully writing and not searching for answers.

The procedure of our study was strictly based on existing studies (Clapham, 1997). The divergent thinking training procedure refers to “Osborn’s checklist” in the method of conceptual skills proposed by Clapham (1997). To meet the actual situation of the COVID-19 pandemic and match the operation procedures of the control group, this study simplified Osborn’s checklist and defined that the theme of divergent thinking training was “Please write down 10 novel functions for the mask.”

In this study, the General Self-Efficacy Scale (GSES) and the Depression-Anxiety-Stress Scale (from DASS-21) were adopted to evaluate self-efficacy, anxiety, depression, and stress level of middle-school students before and 9 days after the experiment. Since the participants were all middle-school students randomly selected from the same school, the demographic variables were balanced between groups.

#### General Self-Efficacy Scale (GSES)

The Chinese version of the GSES was first used by Zhang and Schwarzer (1995) among freshmen in Hong Kong, China, and was later revised and translated into a simplified version by Wang in 2001. The scale is primarily used to measure the self-confidence of individuals when they encounter difficulties and is scored on a five-point Likert scale, with each item scoring from 1 to 5. Our study selected four items, including “If I try my best, I can always solve the problem,” “I am confident that I can effectively deal with anything unexpected,” “If I make the necessary efforts, I will be able to solve most of the problems,” and “When I am in trouble, I can usually think of some ways to deal with it.” For each item, participants were required to answer 1 = very inconsistent, 2 = relatively inconsistent, 3 = general, 4 = more consistent, and 5 = very consistent. The higher the score, the higher the level of general self-efficacy. In the samples of this study, the internal consistency Cronbach’s alpha coefficients of the scale were 0.817 (T1) and 0.878 (T2).

#### Depression-Anxiety-Stress Scale (From DASS-21)

The Depression-Anxiety-Stress Scale was originally an anxiety, depression, and stress scale compiled by Lovibond and Lovibond (1995), and was later revised into a simplified Chinese version by Gong et al. (2010). The scale was scored on a four-point-Likert scale, with each item scoring from 1 to 4. In our study, three items were selected to evaluate the anxiety subscale: (1) “I was worried about situations in which I might panic and make a fool for myself,” (2) “I was aware of the action of my heart in the absence of physical exertion,” and (3) “I feel scared without any good reason.” To assess the level of depression, the following items were selected: (1) “I do not seem to feel any happiness or comfort at all,” (2) “I do not think I have anything to look forward to in the near future,” and (3) “I feel depressed.” The following items for assessing stress levels were selected: (1) “I find it difficult to start learning on my own initiative,” (2) “I feel I have consumed a lot of energy,” and (3) “I cannot tolerate anything that prevents me from continuing my study.”

For each item, participants were required to answer “1 = not consistent, 2 = sometimes consistent, 3 = often consistent, and 4 = always consistent.” The higher the score, the higher the level of anxiety, depression, and stress. In the samples of our study, the internal consistency Cronbach’s alpha coefficients of the scale were 0.876 (T1) and 0.865 (T2).

## Results

To investigate whether there was a difference between groups before the manipulation, multivariate analysis of variance was conducted, taking groups as independent variables, while gender, self-efficacy, anxiety, depression, and stress at T1 were the dependent variables. The results showed that the total effect of group was not significant, *Wilks’ Lambda* = 0.930, *F*(5,55) = 1.057, *p* = 0.386. As presented in Table 1, further univariate analysis showed that there was no difference between two groups on self-efficacy, anxiety, depression, and stress in T1. We also conducted a chi-square test on the gender distribution of the two groups, and the results showed that there was no significant difference χ^2^ (2) = 0.403, *p* = 0.525. In this paper, the data were analyzed through SPSS 21.

**TABLE 1:** Comparison of the two groups in T1.

Independent variable	Experimental group (*n* = 30)	Control group (*n* = 31)	*F*(1,59)	*p*	η*_*p*_^2^*
	
	*M (SD)*	*M (SD)*			
Self-efficacy	12.67 (0.50)	12.71 (0.49)	0.004	0.951	0.000
Anxiety	5.57 (0.38)	6.55 (0.38)	3.373	0.071	0.05
Depression	5.17 (0.37)	5.84 (0.36)	1.704	0.197	0.028
Stress	6.00 (0.39)	7.00 (0.38)	3.155	0.081	0.051

**FIGURE 2:**
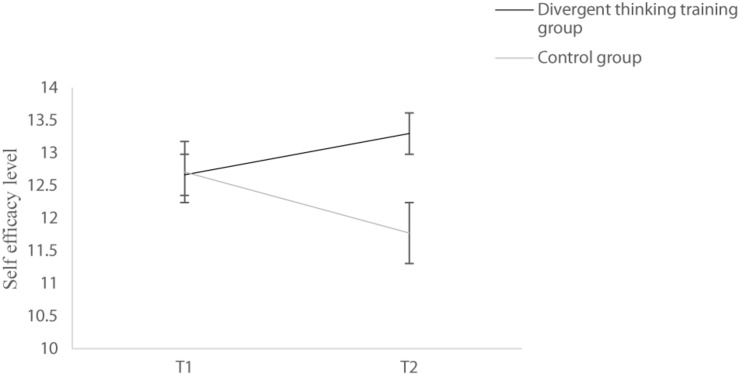
Self-efficacy of two groups before intervention (T1) and 9 days after intervention (T2). The error line represents the standard error.

To analyze the impact of divergent thinking training on self-efficacy, anxiety, depression, and stress, we first used the group (divergent thinking training group vs. control group) as the between-subject variable while the measurement (T1 vs. T2) as the within-subject variable to conduct the repeated measurement analysis of variance. In terms of self-efficacy, it was found that the main effect of group was not significant *F*(1,59) = 1.072, *p* = 0.305, and the main effect of measuring time was not significant either, *F*(1,59) = 0.277, *p* = 0.601. However, the interaction was significant, *F*(1,59) = 7.458, *p* = 0.008, η*_*p*_*^2^ = 0.112. As shown in Figure 2, further simple effect analysis showed that the self-efficacy of the control group in the post-test was lower than that of the pretest, *F*(1,59) = 5.392, *p* = 0.024, η*_*p*_*^2^ = 0.084, which did not occur in the experimental group, *F*(1,59) = 2.392, *p* = 0.127.

**FIGURE 3:**
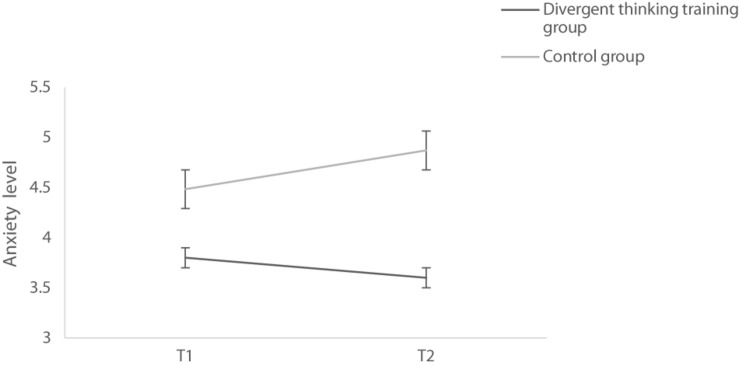
Anxiety of two groups before intervention (T1) and 9 days after intervention (T2). The error line represents the standard error.

In terms of anxiety, it was found that the main effect of group was significant, *F*(1,59) = 6.741, *p* = 0.012, η*_*p*_*^2^ = 0.103, indicating that the level of anxiety in the control group was higher than that of the experimental group. The main effect of time was not significant, *F*(1,59) = 0.845, *p* = 0.362; the interaction was significant, *F*(1,59) = 5.937, *p* = 0.018, η*_*p*_*^2^ = 0.091. Further analysis showed that anxiety of the control group at T1 was higher than that at T2, *F*(1,59) = 5.725, *p* = 0.020, η*_*p*_*^2^ = 0.088. In contrast, the experimental group showed no difference, *F*(1,59) = 1.132, *p* = 0.292. In Figure 3, it can be easily seen that anxiety of the control group increased, while that of the experimental group did not differ between T1 and T2. Meanwhile, we also found that these two groups did not differ on anxiety in T1, *F*(1,59) = 3.373, *p* = 0.071; however, in T2, the control group was more anxious than the experimental group, *F*(1,59) = 9.748, *p* = 0.003,η*_*p*_*^2^ = 0.142. As for depression and stress, neither the main effects of measuring time and group nor the interaction was significant, *ps* > 0.1.

Considering that depression and anxiety (T1: *r* = 0.67, T2: *r* = 0.62) and stress and anxiety (T1: *r* = 0.60, T2: *r* = 0.65) were highly related, we conducted multivariate analysis of covariance to avoid magnification of α error. We analyzed the buffering effect of divergent thinking on anxiety, taking group as the independent variable, self-efficacy, anxiety, depression, and stress in T2 as dependent variables, and gender, anxiety, depression, and stress in T1 as covariates. The results showed that after anxiety, depression, and stress in T1 were controlled, the group effect was significant, *Wilks’ Lambda* = 0.859, *F*(5,55) = 2.896, *p* = 0.044, η*_*p*_*^2^ = 0.141. Further univariate analysis based on *p* correction (*p* = 0.05/2) showed that significant group effects were found on anxiety, *F*(1,55) = 7.534, *p* = 0.008 < 0.025, η*_*p*_*^2^ = 0.120. Therefore, after controlling gender, grade, as well as scores on anxiety, depression, and stress at T1, the anxiety score of the experimental group was significantly lower than that of the control group. However, no significant group effect was found in depression and stress response *F*(1,55) = 0.555, *p* = 0.459, *F*(1,55) = 1.062, *p* = 0.307, which confirmed the results of the repeated measurement analysis of variance.

## Discussion

In our research, we aim to help teenagers solve their psychological problems during the COVID-19 pandemic. Specifically, we conducted a 9-day divergent thinking training and found that the experimental group’s level of anxiety was lower and self-efficacy was higher than that of the control group; meanwhile, stress and depression were not impacted by our manipulation.

### The Impact of Divergent Thinking on Self-Efficacy

Consistent with previous literature (Saigh et al., 1995; Ginzburg et al., 2003), the results showed that self-efficacy of the control group was lower at T2 than T1. Benight and Bandura (2004) proposed that an individual’s appraisal of threat reflected both the nature of the activating stimulus and her/his coping ability. This suggests that once a person feels control over the threat, he/she would not be frightened by it. Disasters place people in situations where they experience a sense of helpless, and lower their self-efficacy by causing death of family members and friends and damage of properties. Besides cognitive appraisal of external stimulus, Alexander and Ward (2018) mentioned that people in danger exhibited some physiological responses such as sweat and pains, which could be viewed as a sign of poor coping capability by individuals, thereby leading to a decrease in their self-efficacy. As we know, COVID-19 is highly infectious and hard to cure, and it has caused a decrease in self-efficacy among people including our participants, due to its wide range of impact and serious destruction.

In contrast, self-efficacy of the experimental group maintained after 9-day divergent thinking training, which confirmed the effectiveness of our manipulation. As mentioned above, self-efficacy is an appraisal of one’s ability to cope with external stimuli. A person with high self-efficacy tends to perceive threat as benign, ruminate less, and stay away from emotional distress (Benight and Bandura, 2004). In contrast, emotional arousal can impair people’s self-efficacy, since people experiencing strong emotions are more likely to exhibit tension and anxiety when facing threat (Bandura, 1977). Accordingly, Bandura (1977) proposed that self-efficacy could be improved by eliminating emotional arousal to threat. He also mentioned that systematic desensitization was especially effective in reducing fear and enhancing self-efficacy, which presented scary things when people were relaxed (Wolpe et al., 1973). In our research, participants in the experimental group were required to list functions for mask. Mask initially represented a threatening thing due to its association with COVID-19 virus, and thinking of it spontaneously induced a sense of scare. As manipulation went on, continuous exposure to mask lowered participants’ arousal to it, which helped restore self-efficacy. It is particularly worth mentioning that the self-efficacy of the experimental group did not increase; rather, it ceased decreasing. Attempting to lower participants’ perception of threat associated with masks was an indirect way to cope with the COVID-19 pandemic, compared to more active preventive behaviors like hand washing and social distancing (Kebede et al., 2020; Tabernero et al., 2020). Nevertheless, our manipulation did lower participants’ fears, which kept their mental state from deteriorating.

### The Impact of Divergent Thinking on Emotions

Parallel with previous literature (Akbari Chermahini and Hommel, 2012; Liknaitzky et al., 2018), our results showed a decrease in anxiety of experimental group, confirming the positive effect of divergent thinking on emotion. As indicated in Akbari Chermahini and Hommel (2012), performing divergent thinking training might boost dopamine level, which triggers positive emotions.

Generally, a decrease in anxiety of experimental group can be attributed to two aspects. First, according to Terror management theory (Greenberg et al., 1986; Pyszczynski et al., 1990), anxiety is induced when people are aware of mortality and vulnerability facing external threats. Being conscious of self-efficacy, which reaffirms an individual’s values and significance, can help lower anxiety induced by self-awareness. Accordingly, as self-efficacy maintained for the experimental group, it is unsurprisingly to see a decrease in anxiety accompanied. Second, ruminative thought, paying attention to negative emotions repeatedly after experiencing stressful events, predicts negative emotions such as anxiety and depression (Nolen-Hoeksema, 2000; Wilkinson et al., 2013; Ruscio et al., 2015). Ruminators keep searching for answers about why negative events happen to them and stay alert to environment around, which contributes to anxiety (Nolen-Hoeksema, 2000). Associative process, pointed out by Bar (2009), could reduce anxiety in two ways. First, Nolen-Hoeksema (2000) attributes people’s anxiety to their uncertainty about question they are searching for, while association enables people to predict and minimize such uncertainty, thereby decreasing anxiety. Second, association allows ruminators to divert their attention away from indulging on negative aspects of experience. In our study, we asked participants in experimental group to list functions for mask, which broadened their thoughts and interrupted rumination. Moreover, attaching various functions to mask allowed participants to convert negative information associated with mask into positive, thus instilling a sense of optimism into them and decreasing anxiety.

Unexpectedly, divergent thinking training exerted no effect on depression or stress. According to findings of Liknaitzky et al. (2018), though divergent thinking is correlated with depression, stimulus included in divergent thinking program should be designed appropriately to guarantee its relation with depressotypic thinking. This might be the reason why depression did not decrease in our study, since our training program is a standard form of divergent thinking task, which does not target a specific emotion. Nevertheless, our results were in line with some literature (Morgan and Harris, 2015; Duan et al., 2020), which show a positive effect of manipulation on anxiety but not stress or depression. This divergent result might lie in different natures among these three variables. Though highly correlated, depression, stress, and anxiety are distinct indicators of emotion (Lovibond and Lovibond, 1995; Moscati et al., 2016). As Lovibond and Lovibond (1995) indicated, a person exhibiting depression has low self-esteem and decreased interest in pursuing goals in life. However, anxiety reflects how a person perceives a threat, and stress indicates how easily a person can become frustrated. COVID-19, as a highly infectious virus, concerns the general public rather than targets a specific person. Accordingly, people are likely to be more sensitive on an anxiety index rather than on a depression or stress index.

### Theoretical Implications

Our research contributes to existing studies in several ways.

First, our study confirms the positive impact of divergent thinking on emotion (Akbari Chermahini and Hommel, 2012; Liknaitzky et al., 2018). In addition, Akbari Chermahini and Hommel (2012) demonstrated how divergent thinking task positively affects general emotions, and our results expand their findings by showing how divergent thinking training contributes to the alleviation of anxiety specifically. However, depression and stress did not decrease after manipulation, despite of confirmed correlation between depression and divergent thinking (Liknaitzky et al., 2018). Thus, researchers need to identify different mechanisms underlying these three variables, and more studies need to be conducted to examine the effect of divergent thinking on different components of emotions.

Second, previous studies have mainly focused on how self-efficacy influences divergent thinking (Kharkhurin, 2017; Puente-Díaz and Cavazos-Arroyo, 2017), and our research extends those findings by showing the positive effect of the latter on the former, implying a reciprocal relationship between divergent thinking and self-efficacy. According to Bandura’s social cognitive theory, lowering the perception of threat allows for an increase in self-efficacy, and the core of divergent thinking training—come up with functions for certain items—could lead to change in one’s perception of external stimulus.

Third, as part of creativity training, divergent thinking could enhance general self-efficacy, rather than merely promote creative self-efficacy in the field of creativity (Mathisen and Bronnick, 2009; Byrge and Tang, 2015; Meinel et al., 2019). In our research, we chose divergent thinking training rather than creativity training as the manipulation; however, we believe creativity training could be effective in promoting self-efficacy and lowering anxiety. Since creativity training is comprised of more diverse training programs (Birdi, 2007; West et al., 2012; Fink et al., 2015), it might play a role in lowering depression and stress, which has not been achieved in our study.

### Practical Implications

The outbreak of the COVID-19 pandemic has caused physical and mental illness for many people (National health commision of the People’s Republic of China, 2020a) and has triggered psychological hazards such as anxiety and depression to those uninfected (Mamidipalli et al., 2020; Mohindra et al., 2020). Therefore, it is of great practical significance to help people cope with psychological problems surrounding the pandemic.

The results of our study are of great practical significance to middle-school students who have experienced a decrease in self-efficacy and an increase in anxiety during the COVID-19 pandemic.

First, our manipulation needs no requirement for time or place—teenagers can be trained whenever necessary. This implies important practical values, since most people have been required to stay at home during the pandemic, conducting complex training programs becomes impossible. Furthermore, divergent thinking training has a standard form (i.e., generation of functions for items), which allows for efficient manipulation and minimal random errors.

Second, we prove that people’s negative mental state could be alleviated by disrupting negative associations with things that scare them. Compared to adults, teenagers have fewer coping strategies for emergency situations and are more vulnerable to mental illness (Garnefski et al., 2002), due to limited experience. Thus, equipping teenagers with more positive thoughts effectively protects their self-efficacy and gives them confidence to cope with unfamiliar circumstances.

Third, our results show that divergent thinking can lead to a decrease in anxiety but not stress or depression. This highlights that despite of high correlation, anxiety, depression, and stress are distinct variables, and they are sensitive to different interventions. Accordingly, practitioners should adopt appropriate intervention programs based on which negative emotion induced in disasters or emergency situations.

### Limitations and Suggestions for Future Research

Although our study provides valuable evidence for the impact of divergent thinking training on self-efficacy and anxiety, some limitations need to be addressed. First, due to the COVID-19 pandemic, our training was conducted online, randomly selecting a middle school in Wuhan and recruiting volunteers. Therefore, the external validity of results remained in doubt, since the severity of the COVID-19 pandemic is different across regions. Specifically, Wuhan has been affected more seriously than other cities, leading to more PTSD symptoms in people. Therefore, we suggest that future researchers conduct training in other regions to determine if the results can be replicated. Also, since all participants are from a middle school, we recommend researchers conduct studies in other age group. Second, due to the isolation policy during the COVID-19 pandemic, we merely conducted online training which raised difficulties for us to hold some variables constant (e.g., social support and physical activity). Thus, we recommend that future researchers adopt face-to-face training and strictly control variables that might impact anxiety, depression, stress, and self-efficacy. Third, though we chose masks as materials used in divergent thinking training, the whole program is a standard form of divergent thinking task. Accordingly, this program might have effect on some emotions but not others, since it was not designed to target specific one. In order to eliminate one specific negative emotion, more elaborate divergent thinking programs need to be designed. Fourth, in order to prevent the influence of repeated measures, we conducted our training immediately after the pretest and did not conduct a middle test. We recommend that future researchers conduct the test three times to obtain more precise results. In addition, as the effect size of self-efficacy (η*_*p*_*^2^ = 0.112) and anxiety (η*_*p*_*^2^ = 0.091) is relatively small, we suggest that practitioners combine divergent thinking training with other trainings to support people in disaster and rebuild their mental health.

## Conclusion

Our work contributes to existing literature on divergent thinking by examining how it impacts self-efficacy and emotions. More specifically, our results reveal that divergent thinking training effectively buffers against decrease in self-efficacy and increase in anxiety among teenagers. Practically, considering that most teenagers are currently under the threat of the COVID-19 pandemic, our findings offer an effective way to enable them to cope with a negative mindset.

## Data Availability Statement

The raw data supporting the conclusions of this article will be made available by the authors, without undue reservation.

## Ethics Statement

The studies involving human participants were reviewed and approved by the Ethics Committee of the Center for Studies of Social Psychology at Central China Normal University (CSSP-2020016). The patients/participants provided their written informed consent to participate in this study.

## Author Contributions

BZ and FW contributed to the design of the study. QW, YQ, and YD organized and analyzed the database, and wrote the different sections of the manuscript. All authors contributed to manuscript revision and read and approved the submitted version.

## Conflict of Interest

The authors declare that the research was conducted in the absence of any commercial or financial relationships that could be construed as a potential conflict of interest.
