# Corrigendum: Impact of Divergent Thinking Training on Teenagers' Emotion and Self-Efficacy During the COVID-19 Pandemic

**DOI:** 10.3389/fpsyg.2021.686118

**Published:** 2021-04-19

**Authors:** Bin Zuo, Qi Wang, Yalan Qiao, Yu Ding, Fangfang Wen

**Affiliations:** Department of Psychology, Center for Studies of Social Psychology, Central China Normal University, Wuhan, China

**Keywords:** divergent thinking, COVID-19, self-efficacy, emotion, teenagers

In the original article, there was an error. An author name was incorrectly spelled as Lan Y. Qiao. The correct spelling is Yalan Qiao.

The authors apologize for this error and state that this does not change the scientific conclusions of the article in any way. The original article has been updated.

