# Supplementary material for: Impact of Divergent Thinking Training on Teenagers’ Emotion and Self-Efficacy During the COVID-19 Pandemic
Source: Front Psychol. 2021 Mar 19;12:600533. doi: 10.3389/fpsyg.2021.600533 (PMC8017284; doi:10.3389/fpsyg.2021.600533)
Supplement: Supplementary file 2 [file Table_2.DOCX]

# Creativity performance

The scale used to measure creativity performance is the Runco Ideational Behavior Scale (RIBS), which was compiled by Runco et al. and revised by Zhang. In this study, nine items were selected to measure creativity performance, which were divided into three dimensions: fluency, originality and flexibility. Fluency consists of three items: "when seeing white clouds or shadows, I imagine what they are based on their shapes", "when seeing a pattern, I have some associations based on its shape" and "I suddenly want to draw something." Originality consists of three items: "when solving mathematical problems, I try different approaches." and "I often change my user name and password" and "I have some inventive ideas." Flexibility consists of three items: "I ponder over a problem from a different perspective", "Although someone tells me how to do one thing, I will come up with other different ways" and "I have contradictory ideas".

The scale was scored on Likert5 points（Never = 1, rarely = 2, sometimes = 3, often = 4, always = 5）, and participants completed it based on their true thoughts. The higher the score, the higher the level of creativity. In our study, the Cronbach's α coefficients of the overall scale were 0.773(T1) and 0.801(T2)，and the Cronbach's α coefficients of each subscale on fluency, originality and flexibility were 0.778, 0.442, 0.678 (T1) and 0.781, 0.589, 0.625（T2）respectively. Taking the group (Divergent thinking group vs. Control group) as the inter-subject variable and the measurement time (T1 vs. T2) as the intra-subject variable, the repeated measurement variance analysis was conducted on fluency, originality and flexibility. The results showed that neither the main effect of group and measurement time, nor the interaction between them were significant, *p*s>0.1.
